# Comparison of Treadmill Gait Between a Pediatric-Aged Individual With *SYNGAP1*-Related Intellectual Disability and a Fraternal Twin

**DOI:** 10.3389/fnhum.2022.918918

**Published:** 2022-06-22

**Authors:** Charles S. Layne, Christopher A. Malaya, David R. Young, Berhard Suter, Jimmy L. Holder

**Affiliations:** ^1^Department of Health and Human Performance, University of Houston, Houston, TX, United States; ^2^Center for Neuromotor and Biomechanics Research, University of Houston, Houston, TX, United States; ^3^Center for NeuroEngineering and Cognitive Science, University of Houston, Houston, TX, United States; ^4^Blue Bird Circle Rett Center, Texas Children’s Hospital, Houston, TX, United States; ^5^Baylor College of Medicine, Houston, TX, United States; ^6^Jan and Dan Duncan Neurological Research Institute at Texas Children’s Hospital, Houston, TX, United States; ^7^Division of Neurology and Developmental Neuroscience, Department of Pediatrics, Baylor College of Medicine, Houston, TX, United States

**Keywords:** neurodevelopmental disorders, walking, biomechanical phenomena, movement disorders, non-linear dynamics (NLD)

## Abstract

*SYNGAP1*-related Intellectual Disability (*SYNGAP1*-ID) is a rare neurodevelopmental condition characterized by profound intellectual disability, gross motor delays, and behavioral issues. Ataxia and gait difficulties are often observed but have not yet been characterized by laboratory-based kinematic analyses. This investigation identified gait characteristics of an individual with *SYNGAP1*-ID and compared these with a neurotypical fraternal twin. Lower limb kinematics were collected with a 12-camera motion capture system while both participants walked on a motorized treadmill. Kinematic data were separated into strides, and stride times calculated. Sagittal plane hip, knee, and ankle joints were filtered and temporally normalized to 100 samples. Minimum and maximum joint angles, range of motion (ROM) and angular velocities were obtained for each joint by stride and averaged for each participant. ROM symmetry between left and right joints was also calculated. Discrete relative phase (DRP) was used to assess coordination and variability between joints within a single limb and compared across limbs. Phase portraits were calculated by joint, and their areas were computed with a MATLAB script. Statistical parametric mapping (SPM) was used to assess differences in joint angle waveforms between participants. P1, the individual with *SYNGAP1*-ID, displayed significantly reduced stride times relative to the fraternal twin, i.e., P2. A majority of minimum, maximum angles, ROMs, and angular velocities were significantly different between P1 and P2. Phase portrait areas were consistently less in P1 relative to P2 and there were differences in knee and ankle symmetries. DRP showed no differences between individuals, suggesting that P1’s coordinative events remained similar to those observed during neurotypical gait (P2). SPM revealed significant differences between the left and right legs at the knee and ankle joints of P1 while P2 joint left and right waveforms were nearly identical for all joints. Additionally, SPM revealed there were significant differences between P1 and P2 for all joints. This investigation identified several major gait features of an individual with *SYNGAP1*-ID and provided a comprehensive characterization of these features by utilizing both linear and non-linear analyses. While limited in generalizability, this report provides a strong quantitative appraisal of gait in an individual with *SYNGAP1*-ID as well as an analysis pathway for future investigations.

## Introduction

Motor deficits, especially gait abnormalities, are understudied, but common, phenotypes associated with neurodevelopmental disorders (Shetreat-Klein et al., 2014; Colizzi et al., 2020). Functional physical deficits result in impaired quality of life for children with neurodevelopmental disorders including those due to monogenic lesions (Bolbocean et al., 2022). Despite this, quantitative evaluations of gait are rarely performed for neurodevelopmental disorders especially as compared to frequent evaluations through neuroimaging, electroencephalography, and neurocognitive testing.

*SYNGAP1*-related Intellectual Disability (*SYNGAP1*-ID) is a rare condition that is characterized by global developmental delays and often accompanied by epilepsy and autism (Squire et al., 1990; Hamdan et al., 2011; Holder et al., 2019; Jimenez-Gomez et al., 2019; Vlaskamp et al., 2019). The prevalence of *SYNGAP1*-ID has not been clearly established, however, the Syngap Global Network has identified 1,055 individuals worldwide as of 2022. Individuals with *SYNGAP1*-ID often have severe to profound intellectual disability, gross motor delays, and features of autism spectrum disorder (ASD) (Squire et al., 1990; Hamdan et al., 2011; Holder et al., 2019; Jimenez-Gomez et al., 2019; Vlaskamp et al., 2019) that are most frequently due to *de novo* autosomal dominant loss-of-function or missense mutations in the *SYNGAP1* gene. Many individuals also have excessive aggression, frequent tantrums, fluctuating moods and sleep difficulties. Other features that are often present and associated with movement control problems include hypotonia, ataxia, and tremor. In a recent study of 57 individuals with *SYNGAP1*-ID mutations, over 50% exhibited ataxia and gait difficulties (Vlaskamp et al., 2019). Movement control problems impair the performance of many activities of daily living as well as prevent consistent exercise for physical fitness.

Several investigators have reported individuals with *SYNGAP1*-ID have gait abnormalities; these are generally described as ataxic, wide-based (Parker et al., 2015; Vlaskamp et al., 2019) or clumsy and unstable (Mignot et al., 2016; Prchalova et al., 2017). However, a review of the literature failed to identify any reports concerning specific parameters that have been associated with the gait of individuals with *SYNGAP1*-ID. Identifying gait characteristics in individuals with *SYNGAP1*-ID is important as gait disorders are associated with significantly increased rates of falling and associated injury. This is particularly important for those with *SYNGAP1*-ID, as approximately 75% of these patients exhibit an increased pain threshold (Vlaskamp et al., 2019). In fact, Vlaskamp et al. (2019) reported that some patients in their study failed to respond to cuts, a fractured bone or an object lodged in their foot. This increased pain threshold—in combination with intellectual disability—could increase the risk of fall-related injury due to an individual’s inability to perceive the potential consequences of failing to safely navigate through their environment (Writzl and Knegt, 2013; Parker et al., 2015; Vlaskamp et al., 2019). Characterizing the gait parameters of those with *SYNGAP1*-ID can be used as a component of an evaluative process to determine the degree of disability as well as track potential progress resulting from therapeutic or pharmacological interventions. The objective of this work was to characterize the gait of an individual with *SYNGAP1*-ID using both linear and non-linear analyses. These techniques provide complimentary information, resulting in a more comprehensive understanding of the unique mobility challenges of this population. This report is the first to provide a quantitative assessment of gait in an individual with a *SYNGAP1*-ID pathogenic mutation compared to his fraternal neurotypical twin.

## Materials and Methods

### Study Participants

Two individuals participated in this investigation. One male with *SYNGAP1*-ID and his healthy female twin were evaluated during treadmill walking. The Institutional Reviews Boards of the University of Houston (00000855) and Baylor College of Medicine (H-35835) approved all procedures. The parents provided written informed consent for both participants.

### Clinical Characteristics of Participants

The affected male subsequently referred to as Participant 1 (P1) was 9 years old at the time of gait assessment, weighed 31.8 kg (68% tile) and was 132.1 cm tall (33% tile). He had a history of global developmental delay including walking independently at 22 months and first spoken word between 3 and 4 years of age. At the time of assessment, he could walk without orthotics, run, walk up and down stairs, and jump all independently. He was not taking medications that would be expected to interfere with his movement coordination.

P1’s neurotypical female twin [Participant 2 (P2)] weighed 29.9 kg (50% tile) and was 135.9 cm tall (60% tile) at time of testing. P2 had no history of developmental delay and her neurologic examination was normal.

### Whole Exome Sequencing in P1

Whole exome sequencing (WES) of P1 was performed on a clinical basis. WES revealed a *de novo* loss-of-function mutation in *SYNGAP1* with c.3718 C > T in NM_006772 (p. R1240X). No other pathogenic variants were identified.

### Participation Preparation

Prior to walking on the motorized treadmill, the participants were fitted with infrared reflective markers placed bilaterally on the heel, 1st metatarsophalangeal joint, lateral malleolus, and shank, as well as the lateral knee and anterior and posterior anterior superior iliac spine (hip). An overhead harness was used to prevent any potential falls but did not provide postural support during walking. Prior to data collection, the participant’s preferred walking speed was determined. Preferred walking speed was found by initiating walking at 0.3 m/s and increasing treadmill speed by 0.1 m/s every 20 s until P1 began to display signs of discomfort such as vocalizations, facial/hand gestures or the parents decided the participant had reached his maximal walking speed. The same procedure was followed for P2. Once maximal speeds had been determined, the treadmill speed was set at 0.2 m/s slower than the identified maximum. Prior to data collection, the participants walked for 2 min at their preferred speed to acclimate to the treadmill. Layne et al. (2017) provides additional details regarding the collection procedures.

### Data Collection and Processing

The participants walked on a split-belt motorized treadmill (Bertec^®^) which contained embedded force plates under each belt. Both participants completed one 45 s trial of walking, which reflected the amount of time P1 was willing to consistently walk. Data were collected at 100 Hz using a Vicon^®^ 12-camera motion capture system and lower limb joint kinematics were obtained.

Kinematic data were filtered using a 2nd order Butterworth low-pass filter with a 6 Hz cutoff frequency using a custom MATLAB^®^ script. Sagittal plane joint angles were calculated for the hip, knee, and ankle of both legs. Heel strikes were identified as the minimum positions of the heel across the waveform; joint angles between consecutive ipsilateral heel strikes were used to partition the waveform into individual strides. The length of each individual partition was used to calculate stride time. Each stride was then time normalized to 100 samples such that the moment of heel strike was represented as time zero for each stride, of each joint. A custom MATLAB script was used to identify minimum and maximum angular values for each joint and stride and used to calculate total range of motion (ROM). Symmetry indices for ROM between the left and right side and for each joint and participant were calculated using the following formula (Hsu et al., 2003).


$$\text{S}\,y\,m\,m\,e\,t\,r\,y\,I\,n\,d\,e\,x=1-\frac{\mathrm{Lesser}\,\mathrm{Angle}}{\mathrm{Greater}\,\mathrm{Angle}}$$

A SI of 0 reflects perfect symmetry between the two limbs. Peak angular velocities for each joint and each stride were also obtained.

Using a variety of linear and non-linear techniques can more fully characterize gait compared to outcomes derived from only one or the other technique. To explore coordination patterns and their variability between joints within a single limb, discrete relative phase (DRP) values were obtained. DRP evaluates the timing between two kinematic events by calculating a discrete phase angle at the specific time points of the events (Hamill et al., 2012). DRP provides information regarding how synchronously the two joints are moving at important moments within a gait cycle. DRP values were computed between the events of peak knee flexion and peak hip extension, peak knee flexion and peak ankle plantar flexion. Means, standard deviations, and medians were calculated for each of the above variables for each participant. Although the data were normally distributed, Levene’s tests revealed the assumption of equality of variance was not met by many variables. In those cases, the Mann-Whitney *U*-test was used to explore potential differences between P1’s and P2’s variables. When the assumption of equality of variance was met, *t*-tests for independent samples were used. Angular position and velocity of each joint were used to develop phase portraits and the area of the portraits were obtained using a custom MATLAB script. Phase portraits provide information regarding the coordination and control associated with movement of a particular joint.

Finally, statistical parametric mapping (SPM) was used to assess potential differences in joint angle waveforms between P1 and P2 (Robinson et al., 2015). In the present study, 22 strides were imported into MATLAB and analyzed. SPM computes the conventional univariate t-statistic between the two mean waveforms as calculated at each sample and identifies statistically significant differences between waveforms. Therefore, SPM provides companion information to the information gained by employing more traditional measures such as identifying single maximum or minimum values within a joint angle waveform. This technique was also used to evaluate potential differences between the left and right joint waveforms for each participant. These results also provide additional information regarding the degree of symmetrical behavior between the two limbs.

## Results

**FIGURE 1:**
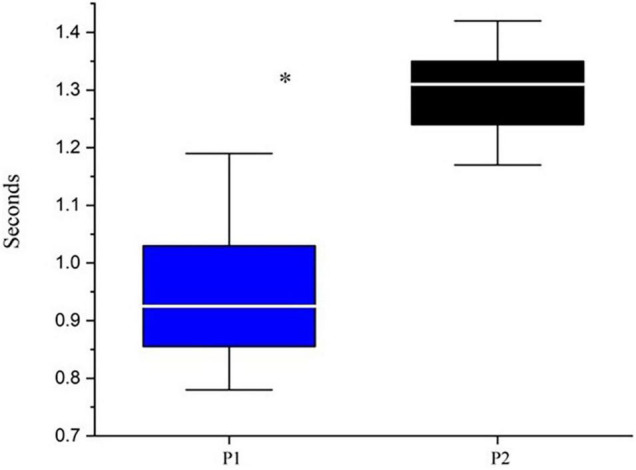
Median and range of stride times. *Indicates *p* < 0.05 based upon Mann-Whitney testing: *U* = 24.5 and *p* < 0.00001. P1 is represented by blue boxes, P2 by black boxes. The white line represents the median.

Following the procedures described in the Methods, preferred walking speed for P1 was 0.6 m/s while P2’s was 0.9 m/s. Figure 1 displays the median and range of stride times for the two participants. P1 had significantly lower strides times and greater variability relative to P2.

**FIGURE 2:**
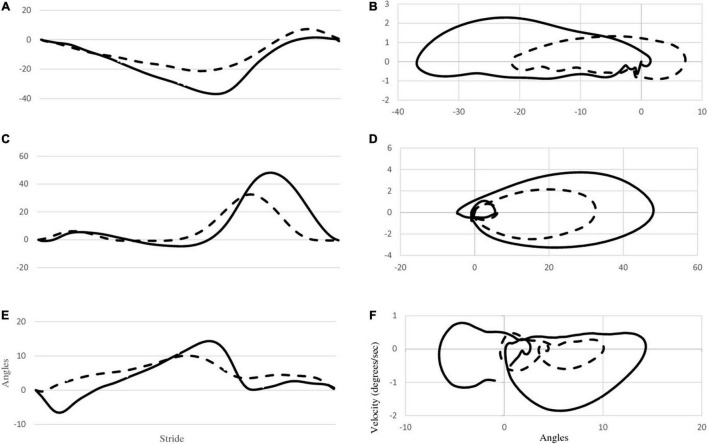
The first column **(A,C,E)** depicts mean joint angle waveforms of the right hip, knee and ankle for both P1 (dashed line) and P2 (solid line). The second column **(B,D,F)** depicts phase portraits for the right hip, knee and ankle for both participants.

**FIGURE 3:**
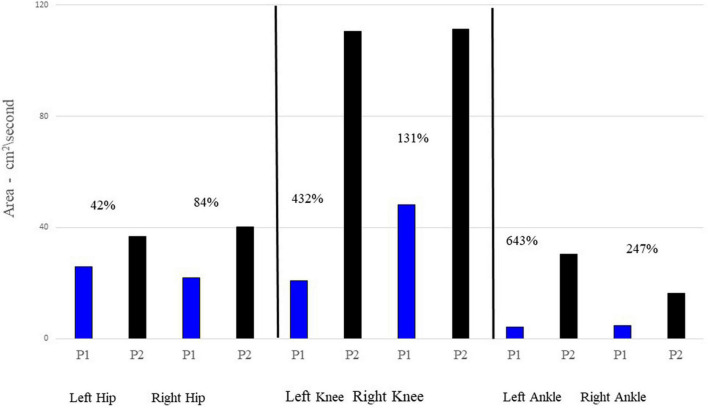
Mean phase portrait area values for each joint and participant. P1’s phase portrait area values are represented by the blue bar graphs; P2 is represented by the black bars. The percentages listed by each comparison provides the percentage increase of P2’s area relative to P1’s area.

Figure 2 provides mean joint angle waveforms and phase portraits for both participants. It can be observed that although there are amplitude differences in the joint angles, the shape of the waveforms are generally similar between the two participants. However, the phase portraits provide information about the control of lower limbs and reflect significant differences between P1 and P2. Mean phase portrait area values are presented in Figure 3 for all joints and both participants.

Table 1 presents medians as well as mean and standard deviation values of the left and right minimum and maximum joint angles as well as total ROM. The Mann-Whitney test and U statistic was used when the assumption the equality of variance was not met, otherwise the *t*-test was used to test for potential differences. There were significant differences between P1 and P2 for every comparison with the exception of the left ankle ROM. In all cases, the median and mean ROM for P1 was less than that of P2.

**TABLE 1:** Median of the minimum and maximum joint angle values, as well as ROM for each joint and both participants.

Joint	*P*	Min	Max	ROM	$\bar{\text{X}}$ min	$\bar{\text{X}}$ max	$\bar{\text{X}}$ ROM
L hip	P1	–27.5	8.2	35.1	–27.7 (7.8)	8.8 (6.9)	36.5 (7.5)
L hip	P2	–35.9	2.9	38.6	–36.5 (3.0)	2.5 (1.8)	39.0 (2.5)
L hip	P1 vs. P2	U = 92 p < 0.000	U = 123 p < 0.000	U = 192 p < 0.036			
L knee	P1	–22.4	25.0	41.2	–20.8 (14.6)	23.9 (19.8)	46.7 (18.1)
L knee	P2	–3.5	50.3	54.6	–4.2 (3.5)	50.1 (5.00)	54.4 (3.9)
L knee	P1 vs. P2	U = 87 p < 0.000	U = 77 p < 0.000	U = 163 p < 0.007			
L ankle	P1	–2.8	24.7	27.5	–3.7 (4.7)	23.9 (8.9)	27.7 (8.4)
L ankle	P2	–6.6	21.1	30.0	–8.3 (4.3)	21.6 (6.1)	29.9 (5.7)
L ankle	P1 vs. P2	U = 22 p < 0.000	U = 1 p < 0.000	U = 274 p = 0.624			
R hip	P1	–26.9	8.8	34.8	–25.2 (8.3)	10.1 (7.2)	35.3 (7.6)
R hip	P2	–38.1	3.5	42.0	–37.9 (6.2)	3.8 (3.1)	41.7 (5.0)
R hip	P1 vs. P2		U = 158.5 p < 0.000		t = –6.0 p < 0.000		t = 3.6 p < 0.000
R knee	P1	–8.9	39.9	47.1	–8.8 (5.0)	38.7 (10.5)	47.4 (10.1)
R knee	P2	–6.3	50.7	55.1	–6.8 (5.2)	50.3 (6.4)	57.1 (3.9)
R knee	P1 vs. P2		U = 90 p < 0.000	U = 97 p < 0.001	t = 1.3 p < 0.021		
R ankle	P1	–1.8	11.6	14.1	–2.3 (2.5)	11.7 (3.0)	14.0 (2.5)
R ankle	P2	–7.5	14.0	22.1	–8.1 (2.9)	14.8 (4.0)	22.9 (4.3)
R ankle	P1 vs. P2			U = 10 p < 0.000	t = 7.7 p < 0.000	t = 3.1 p < 0.003	

*The results were either analyzed by the Mann-Whitney test or t-test and associated p-value are also included.*

Table 2 highlights the differences between P1 and P2 across all joints. All maximum velocities of P2—with the exception of the left ankle—were greater than those of P1.

**TABLE 2:** Median and mean maximum angular velocities and standard deviations in degrees per second for each joint and participant.

Joint	Participant	Max	$\bar{\text{X}}\,\text{ma}\,x$
Left hip	P1	1.9	2.2 (1.00)
Left hip	P2	2.3	2.3 (0.35)
Left hip	P1 vs. P2	*U* = 99, *p* < 0.000	
Left knee	P1	2.3	3.4 (3.73)
Left knee	P2	3.7	3.6 (0.36)
Left knee	P1 vs. P2	*U* = 124, *p* < 0.000	
Left ankle	P1	3.0	3.5 (1.56)
Left ankle	P2	1.8	2.1 (1.12)
Left ankle	P1 vs. P2	*U* = 95, *p* < 0.000	
Right hip	P1	2.0	2.0 (0.58)
Right hip	P2	2.8	2.7 (0.44)
Right hip	P1 vs. P2		*t* = 5.0, *p* < 0.000
Right knee	P1	2.7	2.6 (0.75)
Right knee	P2	4.4	4.3 (0.68)
Right knee	P1 vs. P2		*t* = 7.5, *p* < 0.000
Right ankle	P1	0.7	0.8 (0.23)
Right ankle	P2	1.4	1.4 (0.42)
Right ankle	P1 vs. P2	*U* = 62, *p* < 0.000	

*Mann-Whitney U and t-test values and associated p-values are also included.*

Figure 3 displays the area of the phase portraits for each joint for the two participants. Similar to Figure 2, the areas of P1’s phase portraits are less than P2’s.

**FIGURE 4:**
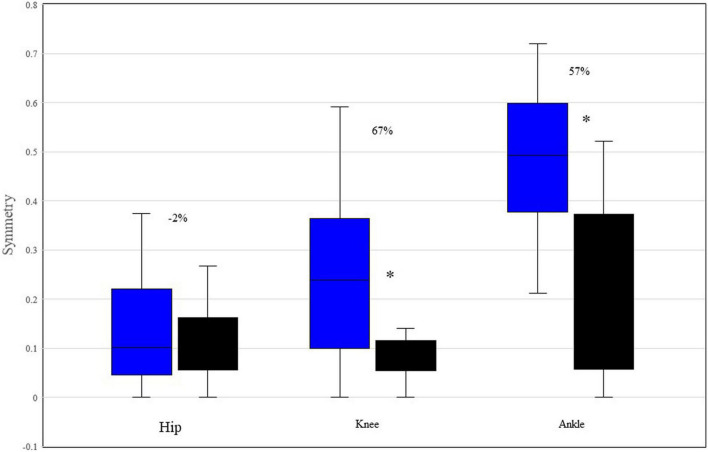
Median and range symmetry values between the left and right ROM of hip, knee, and ankle, for each participant. *Indicates *p* < 0.05 based upon Mann-Whitney testing. The percentages reflect the differences between P1 and P2. P1 is represented by blue boxes, P2 by black boxes.

Figure 4 displays Box and Whisker plot of the median symmetry values between the left and right ROM for each joint and participant. The symmetry of the knee and ankle of P1 is significantly less than that of P2.

Table 3 displays the medians of the discrete relative phase (DRP) values for the two comparisons and both limbs as assessed using Mann-Whitney tests. Interestingly, no DRP differences between the two participants reached statistical significance.

**TABLE 3:** Discrete relative phase (DRP) values for left and right lower limb comparisons.

Comparisons	P1	P2		
	**DRP**	**DRP**	***U***	***P***
Left hip min vs. left knee max	–86.4	–75.6	242	0.638
Right hip min vs. right knee max	–64.8	–64.8	238.5	0.952
Left knee max vs. left ankle min	183.6	241.2	215	0.395
Right knee max vs. right ankle min	216.0	244.8	176	0.187

**FIGURE 5:**
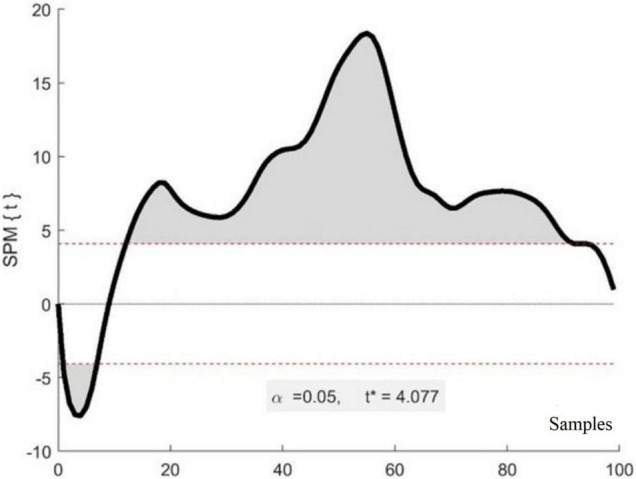
Exemplar results of SPM analysis. The *x*-axis represents a stride composed of 100 samples (heel strike to heel strike) while the *y*-axis represents the *t*-value for each sample of a stride. The shaded areas indicate samples that are significantly different between the two waveforms of the two participants. The * identifies the *t*-value.

Figure 5 is an exemplar figure that shows the SPM comparison between P1 and P2 left knee waveforms and reveals that there are significant differences between the two waveforms within several phases of the gait cycle.

Table 4 displays the results of SPM analyses from comparisons between the left and right joints by participant. For P1, of the 21 phases (7 phases by 3 joints), six (28.6%) displayed significant differences all of which were at the knee. Interestingly, the comparison between the left and right hips and ankles displayed no differences between the waveforms. For the knee, all phases but the terminal swing were significantly different. Conversely, for P2, only the initial swing phase of the knee was significantly different (15.4% of the stride). There were no other comparisons that revealed differences between the left and right waveforms for the three joints for this participant.

**TABLE 4:** The results of SPM left vs. right leg analyses separated by gait cycle phases for each participant.

Gait phase	Loading	MStance	TStance	PSwing	ISwing	MSwing	TSwing
% of stride	0–10	11–30	31–50	51–60	61–73	74–87	88–100
P1 hip (%)	0	0	0	0	0	0	0
P2 hip (%)	0	0	0	0	0	0	0
P1 knee (%)	90	100	100	100	100	28.6	0
P2 knee (%)	0	0	0	0	15.4	0	0
P1 ankle (%)	0	0	0	0	0	0	0
P2 ankle (%)	0	0	0	0	0	0	0

*The seven phases are loading, midstance (MStance), terminal stance (TStance), preswing (PSwing), initial swing (ISwing), midswing (MSwing) and terminal swing (TSwing) (Stöckel et al., 2015).*

Table 5 shows the results of the SPM analyses comparing the times series waveform of each joint between P1 and P2 across the gait cycles. The results are presented as the percent of samples that are statistically significantly different within each gait phase. Of the 42 evaluated phases (6 joints by 7 phases), 23 (54.8%) displayed differences within the phases.

**TABLE 5:** The results of SPM analyses between the same joints of the two participants separated by gait cycle phases (see Table 4’s legend for phase labels).

Gait phase	Loading	MStance	TStance	PSwing	ISwing	MSwing	Tswing
% of stride	0–10	11–30	31–50	51–60	61–73	74–87	88–100
Left hip (%)	60	25	40	100	61.5	0	0
Left knee (%)	90	100	0	0	76.9	100	15.4
Left ankle (%)	50	0	0	0	0	0	0
Right hip (%)	0	0	30	100	100	35.7	0
Right knee (%)	0	30	0	0	30.8	100	38.5
Right ankle (%)	0	90	100	100	69.2	0	0

## Discussion

This report details multiple gait variables associated with treadmill walking of an individual with *SYNGAP1*-related Intellectual Disability (*SYNGAP1*-ID) and his fraternal female twin. The combination of linear and non-linear analysis techniques provided complimentary insights into specific gait parameters that varied significantly from his neurotypical twin.

Figure 1 illustrates that the stride times of P1 were significantly reduced related to his twin. For comparison purposes, P1 mean stride time was 0.96 s; in a study of young walkers conducted by Lythgo et al. (2011), 5-year-old participants had stride times of 0.97 s when asked to walk slowly. These same participants had stride times of 1.27 s when asked to walk at a comfortable pace. In this same study, 9-year-old participants had an average stride time of 1.35 s when asked to walk at a comfortable pace while P2 had stride times of 1.30 s in the current investigation. Thus at least in terms of stride times, P1 resembles walkers younger than his chronological age while those of P2 were more age appropriate.

The most striking finding in this investigation is the reduced joint ROM exhibited by P1. With the exception of the left ankle, all of P1’s ROMs were significantly less than those of P2. This can be seen in Figures 2A,C,E, as well as in Table 1. This reduced ROM was accompanied by significantly reduced peak velocities in all of the joints (Figures 2B,D,F and Table 2). The phase portraits in Figure 2 emphasize the large differences in the magnitude of the coordination patterns within the lower limbs. These figures are supported by multiple comparisons revealing statistical differences between P1 and P2 as displayed in Tables 1, 2 as well as Figure 3. Lower walking speeds are associated with reductions in joint motion (Fukuchi et al., 2019) and the reduced range of P1’s ROMs are consistent with the P1’s reduced stride time and lower preferred walking speed.

Figure 4 indicates that both the knee and ankle of P1 function significantly less symmetrically than those of P2, though there is no difference in hip symmetry. Moreover, given that zero reflects perfect symmetry, P1’s knees and ankles function are notably asymmetrical with regards to their respective ROMs. Conversely, P2 displayed almost universal symmetry with only a single phase (15.4% of the initial swing in the knee joint) indicating that any samples in the left and right joint waveforms were not following the same pattern with similar magnitudes (Table 4). A recent study reported that the symmetry of the hip, knee, and ankle of healthy, young adults displayed 93, 91, and 84% symmetry, respectively between the left and right legs during walking. P1 displayed 89, 75, and 47% symmetry for the hip, knee and ankle, respectively. Conversely, P2 displayed 88, 92, and 78% symmetry for hip, knee, and ankle, respectively. Given that symmetrical behavior between the right and left lower limbs develops early in young walkers and remains consistent both across age and gait speed (Lythgo et al., 2011), our data indicate that P1’s limb behavior is outside of the range for similar aged neurotypical walkers and progressively deteriorates proximally to distally. It has been demonstrated that asymmetrical walking is associated with increased oxygen consumption and energy costs relative to more symmetrical gait (Patterson et al., 2008; Viteckova et al., 2018). Therefore, it is likely that P1’s asymmetrical gait is also less efficient than that of neurotypical individuals. This provides a target area for rehabilitation programs.

Discrete relative phase values (DRPs) quantify the control of two joints during important kinematic events, irrespective of joint motion magnitude. In this investigation, DRP values did not reflect significant differences between gait events for P1 and P2. This indicates that the relationship between the peak events (either peak flexion or extension, depending upon the joint) were similar for P1 and P2. This finding suggests that P1 had similar coordinative features as P2 during important gait events, despite having significant differences in the magnitudes, velocities, and waveforms patterns of those same joints. This finding indicates that the timing of important lower limb gait events is maintained in *SYNGAP1*-ID relative to neurotypical walkers even when overall ROM symmetry is not.

The results of the within-participant SPM analyses presented in Table 4 specifies that throughout the ROM of P1’s left and right hips and ankles, respectively, function similarly. This is reflected in the lack of differences across the seven gait phases for the hip and ankle. This finding reinforces the value of using both 0D and 1D measures. Using only 0D ROM measures (Figure 4), P1’s left and right ankle joints displayed extreme asymmetry but using SPM (Table 4) which assessed potential differences across the entire waveform, no significant differences were identified. Conversely, P1’s knee ROM 0D symmetry measure displayed significant asymmetry and this asymmetry was also observed with the use of SPM.

Despite high symmetry values represented in Table 4 for P1’s hip and ankle, Table 5 indicates that there were significant differences across the waveforms of all the joints, with the exception of the left ankle, when P1 and P2 were compared using SPM. These findings suggest that P1’s overall lower limb coordination is significantly different than his neurotypical twin. Whether this is consistent among all individuals with *SYNGAP1*-ID warrants further investigation.

It is important to note that this investigation only featured a single pair of participants. While this does limit generalization to other individuals with *SYNGAP1*-ID, given the lack of quantitative reports concerning gait parameters associated with this syndrome, we believe the report makes a significant contribution to the literature, both for its findings as well as suggested analysis techniques.

In summary, *SYNGAP1*-ID appears to reduce walking speed, decrease joint ROM and velocity, and significantly alters limb coordination, as well as ROM joint symmetry in the knees and ankles. Despite these differences, there is evidence that hip kinematics and some temporal features of gait remain relatively unchanged. Although not subject to formal evaluation in this investigation, many of the measures indicate that P1 displays significantly increased variability relative to P2. Exploring both intra- and inter-subject variability should be the incorporated in future studies. The present data suggests that rehabilitation programs should focus on activities that increase the ROM for all joints as well as angular velocities. Potentially structured walking programs combined with traditional physical therapy programs may serve to improve the gait of individuals with *SYNGAP1*-ID. Additionally, future studies should consider utilizing a variety of analyses, both linear and non-linear, in order to provide a more comprehensive picture of lower limb motion.

## Data Availability Statement

The raw data supporting the conclusions of this article will be made available by the authors, without undue reservation.

## Ethics Statement

The studies involving human participants were reviewed and approved by the Institutional Reviews Boards of the University of Houston (00000855) and Baylor College of Medicine (H-35835). Written informed consent to participate in this study was provided by the participants’ legal guardian/next of kin.

## Author Contributions

CL was responsible for study conceptualization, data analysis, and writing the text of the manuscript. CM was responsible for data processing and manuscript preparation. DY was responsible for data collection and manuscript review. BS was responsible for study conceptualization and manuscript review. JH was responsible for study conceptualization, manuscript preparation, and review. All authors contributed to the article and approved the submitted version.

## Conflict of Interest

The authors declare that the research was conducted in the absence of any commercial or financial relationships that could be construed as a potential conflict of interest.

## Publisher’s Note

All claims expressed in this article are solely those of the authors and do not necessarily represent those of their affiliated organizations, or those of the publisher, the editors and the reviewers. Any product that may be evaluated in this article, or claim that may be made by its manufacturer, is not guaranteed or endorsed by the publisher.

## References

[B1] Bolbocean C. Andújar F. N. McCormack M. Suter B. Holder J. L. Jr. (2022). Health-Related Quality of Life in Pediatric Patients with Syndromic Autism and their Caregivers. *JADD* 52 1334–1345. 10.1007/s10803-021-05030-8 33937973 PMC8854255

[B2] Colizzi M. Ciceri M. L. Di Gennaro G. Morari B. Inglese A. Gandolfi M. (2020). Investigating Gait, Movement, and Coordination in Children with Neurodevelopmental Disorders: Is There a Role for Motor Abnormalities in Atypical Neurodevelopment? *Brain Sci.* 10:601. 10.3390/brainsci10090601 32887253 PMC7565603

[B3] Fukuchi C. A. Fukuchi R. K. Duarte M. (2019). Effects of walking speed on gait biomechanics in healthy participants: a systematic review and meta-analysis. *Syst. Rev.* 8:153. 10.1186/s13643-019-1063-z 31248456 PMC6595586

[B4] Hamdan F. F. Daoud H. Piton A. Gauthier J. Dobrzeniecka S. Krebs M. O. (2011). De novo SYNGAP1 mutations in nonsyndromic intellectual disability and autism. *Bio psychiatry* 69 898–901. 10.1016/j.biopsych.2010.11.015 21237447

[B5] Hamill J. Palmer C. Van Emmerik R. E. (2012). Coordinative variability and overuse injury. *Sports. Med. Arthrosc. Rehabil. Ther. Technol.* 4:45. 10.1186/1758-2555-4-45 23186012 PMC3536567

[B6] Holder J. L. Jr. Hamdan F. F. Michaud J. L. (2019). “SYNGAP1-Related Intellectual Disability,” in *GeneReviews^®^*, eds Adam M. P. Mirzaa G. M. Pagon R. A. Wallace S. E. Bean L. J. H. Gripp K. W. (Seattle: University of Washington). 30789692

[B7] Hsu A. L. Tang P. F. Jan M. H. (2003). Analysis of impairments influencing gait velocity and asymmetry of hemiplegic patients after mild to moderate stroke. *Arch. Phys. Med. Rehabil.* 84 1185–1193. 10.1016/s0003-9993(03)00030-3 12917858

[B8] Jimenez-Gomez A. Niu S. Andujar-Perez F. McQuade E. A. Balasa A. Huss D. (2019). Phenotypic characterization of individuals with SYNGAP1 pathogenic variants reveals a potential correlation between posterior dominant rhythm and developmental progression. *J. Neurodev. Dis.* 11:18. 10.1186/s11689-019-9276-y 31395010 PMC6688356

[B9] Layne C. S. Lee B.-C. Young D. Knight A. Glaze D. G. Schwabe A. (2017). Methodologies to objectively assess gait and postural control features in Rett syndrome. *RARE J.* 4 1–7.

[B10] Lythgo N. Wilson C. Galea M. (2011). Basic gait and symmetry measures for primary school-aged children and young adults. II: walking at slow, free and fast speed. *Gait Post.* 33 29–35. 10.1016/j.gaitpost.2010.09.017 20971013

[B11] Mignot C. von Stülpnagel C. Nava C. Ville D. Sanlaville D. Lesca G. (2016). Genetic and neurodevelopmental spectrum of SYNGAP1-associated intellectual disability and epilepsy. *J. Med. Genet.* 53 511–522. 10.1136/jmedgenet-2015-103451 26989088

[B12] Parker M. J. Fryer A. E. Shears D. J. Lachlan K. L. McKee S. A. Magee A. C. (2015). De Novo, Heterozygous, Loss-of-Function Mutations in SYNGAP1 Cause a Syndromic Form of Intellectual Disability. *Am. J. Med. Genet. Part A* 167A 2231–2237. 10.1002/ajmg.a.37189 26079862 PMC4744742

[B13] Patterson K. K. Parafianowicz I. Danells C. J. Closson V. Verrier M. C. Staines W. R. (2008). Gait asymmetry in community-ambulating stroke survivors. *Arch. Phys. Med. Rehabil.* 89 304–310. 10.1016/j.apmr.2007.08.142 18226655

[B14] Prchalova D. Havlovicova M. Sterbova K. Stranecky V. Hancarova M. Sedlacek Z. (2017). Analysis of 31-year-old patient with SYNGAP1 gene defect points to importance of variants in broader splice regions and reveals developmental trajectory of SYNGAP1-associated phenotype: case report. *BMC Med. Genet.* 18:62. 10.1186/s12881-017-0425-4 28576131 PMC5457574

[B15] Robinson M. A. Vanrenterghem J. Pataky T. C. (2015). Statistical Parametric Mapping (SPM) for alpha-based statistical analyses of multi-muscle EMG time-series. *J. Electromyogr. Kinesiol.* 25 14–19. 10.1016/j.jelekin.2014.10.018 25465983

[B16] Shetreat-Klein M. Shinnar S. Rapin I. (2014). Abnormalities of joint mobility and gait in children with autism spectrum disorders. *Brain Dev.* 36 91–96. 10.1016/j.braindev.2012.02.005 22401670

[B17] Squire E. N. Jr. Kosisky S. E. Shah A. S. (1990). Delayed-type hypersensitivity skin testing: variance among Trichophyton species extracts. *Allergy* 45 157–158. 10.1111/j.1398-9995.1990.tb00475.x 2316826

[B18] Stöckel T. Jacksteit R. Behrens M. Skripitz R. Bader R. Mau-Moeller A. (2015). The mental representation of the human gait in young and older adults. *Front. Psychol.* 6:943. 10.3389/fpsyg.2015.00943 26236249 PMC4500916

[B19] Viteckova S. Kutilek P. Svoboda Z. Krupicka R. Kauler J. Szabo Z. (2018). Gait symmetry measures: a review of current and prospective methods. *Biomed. Signal Process. Control* 42 89–100. 10.1016/j.bspc.2018.01.013

[B20] Vlaskamp D. R. M. Shaw B. J. Burgess R. Mei D. Montomoli M. Xie H. (2019). SYNGAP1 encephalopathy: a distinctive generalized developmental and epileptic encephalopathy. *Neurology* 92 e96–e107. 10.1212/WNL.0000000000006729 30541864 PMC6340340

[B21] Writzl K. Knegt A. C. (2013). 6p21.3 Microdeletion involving the SYNGAP1 gene in a patient with intellectual disability, seizures, and severe speech impairment. *Am. J. Med. Genet. Part A* 161A 1682–1685. 10.1002/ajmg.a.35930 23687080

